# Is Non-Steroidal Anti-Inflammatory Therapy Non-Inferior to Antibiotic Therapy in Uncomplicated Urinary Tract Infections: a Systematic Review

**DOI:** 10.1007/s11606-020-05745-x

**Published:** 2020-04-08

**Authors:** Matthew R. Carey, Valerie M. Vaughn, Jason Mann, Whitney Townsend, Vineet Chopra, Payal K. Patel

**Affiliations:** 1 University of Michigan Medical School, Ann Arbor, MI USA; 2 Division of Hospital Medicine, Department of Internal Medicine, Michigan Medicine, Ann Arbor, MI USA; 3 Department of Internal Medicine, VA Ann Arbor Healthcare System, Ann Arbor, MI USA; 4 Institute for Healthcare Policy and Innovation, University of Michigan, Ann Arbor, MI USA; 5 Taubman Health Sciences Library, University of Michigan, Ann Arbor, MI USA; 6 Division of Infectious Diseases, Department of Internal Medicine, Michigan Medicine, Ann Arbor, MI USA

**Keywords:** urinary tract infection, systematic review, NSAIDs, antibiotics, antibiotic stewardship

## Abstract

**Background:**

Amid growing antimicrobial resistance, there is an increasing focus on antibiotic stewardship efforts to reduce inappropriate antibiotic prescribing. In this context, novel approaches for treating infections without antibiotics are being explored. One such strategy is the use of non-steroidal anti-inflammatory drugs (NSAIDs) for uncomplicated urinary tract infections (UTIs). Therefore, we conducted a systematic review of randomized controlled trials to evaluate the rates of symptom resolution and infectious complications in adult women with uncomplicated UTIs treated with antibiotics versus NSAIDs.

**Methods:**

We systematically searched PubMed, CINHAL, Scopus, Web of Science Core Collection, EMBASE, and ClinicalTrials.gov from inception until January 13, 2020, for randomized controlled trials comparing NSAIDs with antibiotics for treatment of uncomplicated UTIs in adult women. Studies comparing symptom resolution between groups were eligible. Two authors screened all studies independently and in duplicate; data were abstracted using a standardized template. Risk of bias was assessed using the Cochrane Collaboration tool.

**Results:**

Five randomized trials that included 1309 women with uncomplicated UTI met inclusion criteria. Three studies (1130 patients) favored antibiotic therapy in terms of symptom resolution. Two studies (179 patients) found no difference between NSAIDs and antibiotics in terms of symptom resolution. Three studies reported rates of pyelonephritis, two of which found higher rates in patients treated with NSAIDs versus antibiotics. Between two studies that reported this outcome (747 patients), patients randomized to NSAIDs received fewer antibiotic prescriptions compared with those in the antibiotics group. Three studies were at low risk of bias, one had an unclear risk of bias, and one was at high risk of bias.

**Discussion:**

For the outcomes of symptom resolution and complications in adult women with UTI, evidence favors antibiotics over NSAIDs.

**Prospero:**

CRD42018114133

**Electronic supplementary material:**

The online version of this article (10.1007/s11606-020-05745-x) contains supplementary material, which is available to authorized users.

## INTRODUCTION

Urinary tract infections (UTIs) are common, ranking as the second most frequent indication for antibiotic prescribing in the outpatient setting.^1, 2^ The majority of UTIs are “uncomplicated,” occurring in non-pregnant adult women who are not immunocompromised and have normal genitourinary structure and function.^3^ Each year, 12.6% of women will have a UTI, and half of all women will have at least one UTI by age 32.^4^ The standard of care for uncomplicated UTI is oral antibiotic therapy, which typically leads to rapid symptom resolution and reduces the risk of complications such as pyelonephritis.^5^

In recent years, growing antibiotic resistance has led to efforts to use antibiotics more judiciously. Specifically for uncomplicated UTIs, these initiatives have sought to reduce the amount—either duration or frequency—and spectrum of antibiotics.^6–8^ Interviews with women indicate willingness to delay or avoid antibiotic prescriptions when safe to do so.^9, 10^ Furthermore, some UTIs are self-limited without treatment and thus may not require antibiotic therapy.^11^ For example, a randomized controlled trial comparing nitrofurantoin with placebo for uncomplicated UTI demonstrated spontaneous symptomatic cure or improvement in over half of participants receiving placebo who had UTIs as proven by a combination of symptoms/signs and positive laboratory testing.^12^ While a meta-analysis of placebo-controlled trials found placebo to be inferior to antibiotics for resolution of UTI symptoms,^13^ it is unclear whether non-antibiotic treatment regimens for uncomplicated UTIs may be feasible as an outpatient antibiotic stewardship strategy. For example, cranberry extract was initially considered a potential antibiotic-sparing option; however, subsequent research has not supported its use in the treatment of UTI over antibiotics.^14^

One potential antibiotic-sparing treatment for UTI is non-steroidal anti-inflammatory drugs (NSAIDs) which potentially provides relief from dysuria related to elevated prostaglandin E2 levels.^15, 16^ Targeting symptom relief is particularly attractive as dysuria, frequency, and urgency may be misattributed to UTI when in fact no infection is present (e.g., “urethral syndrome”).^17^ Furthermore, though evidence is mixed, NSAIDs may also have direct antimicrobial properties.^18–21^ Within this context, Bleidorn and colleagues demonstrated in a 2010 randomized pilot study that NSAIDs were non-inferior for improving symptoms as well as preventing relapse and non-serious adverse events when compared with antibiotics for uncomplicated UTI.^22^ Conversely, in 2015, Gágyor and colleagues found treatment with NSAIDs to be less effective compared with treatment with antibiotics for symptom relief and to result in more cases of pyelonephritis.^23^ Similarly, evidence has been mixed for using NSAIDs for UTI in other contexts. While NSAIDs may be less effective than antibiotics for inpatient UTI,^24^ there is a potential role for NSAID therapy as part of antibiotic stewardship efforts that include delayed antibiotic prescriptions.^25^

To better understand whether NSAIDs may help in management of UTI and reduce overall antibiotic prescribing, we performed a systematic review of randomized controlled trials to determine the effect of treatment with NSAIDs versus antibiotics for uncomplicated UTI on symptom resolution, development of infectious complications, and antibiotic prescribing frequency.

## METHODS

We published a protocol (PROSPERO CRD42018114133) and followed the PRISMA (Preferred Reporting Items for Systematic Reviews and Meta-Analyses) recommendations in the conduct of this systematic review.^26^

### Data Sources and Searches

A medical librarian (WT) performed literature searches in the following databases from their inception through January 13, 2020: PubMed, CINHAL (EBSCO), Scopus, Web of Science Core Collection, EMBASE (Embase.com), and ClinicalTrials.gov. Searches for each database included appropriate controlled vocabulary terms when available, combined with keywords such as “UTIs,” “Analgesics,” and “Antibiotics” (Appendix). No search restrictions were placed on publication date, language, or completion status. To ensure inclusion of clinical trials that may have been completed but not yet published, we reviewed the Cochrane Central Register of Controlled Trials website as well. We also performed hand-searched of bibliographies to ensure inclusion of all relevant articles.

### Study Selection

Randomized controlled trials were eligible for inclusion if they compared antibiotics versus NSAIDs for treatment of uncomplicated UTIs. Uncomplicated UTIs were defined as UTIs (diagnosed based on symptoms of urgency, dysuria, frequency, or suprapubic tenderness, with or without dipstick or culture evidence of bacteriuria) in non-pregnant adult women who were not immunocompromised and had no prior urological procedures or hardware. “Adult” was defined as 18 years of age or older for studies conducted in countries that are members of the Organization for Economic Cooperation and Development (OECD). For studies conducted in other countries, a lower age limit of 15 years of age was used after discussion among two authors (MRC, PKP). Two authors (MRC, PKP) independently determined study eligibility. A third author (VV) resolved any differences of opinion between the two primary reviewers. Interrater agreement for study eligibility was assessed using Cohen’s kappa.

### Data Extraction and Quality Assessment

Data were extracted from the included studies by one author (MRC) and verified by another (PKP) using a standardized template. Data extracted included the number and type of patients, definitions of UTI, and outcome measures (time to symptom resolution, number of antibiotic prescriptions, cases of pyelonephritis).

Two authors (MRC and PKP) independently assessed the risk of bias in included trials using the Cochrane Collaboration risk of bias tool.^27^ Studies were only defined as “low risk” if they met the criteria for low risk of bias for all six domains. Studies with missing methodological data were considered to be at unclear risk of bias.

### Data Synthesis and Analysis

The primary outcome of interest was the proportion of individuals with resolution of symptoms by day 3 or day 4 post-randomization. In accordance with existing studies, we chose 30 days post-randomization for assessment of secondary outcomes that included (a) incidence of pyelonephritis and (b) number of antibiotic prescriptions. For all outcomes, we reported the difference in risk with 95% confidence intervals. When data regarding symptom resolution in both groups were available, we calculated the number needed to treat (NNT) with antibiotics versus NSAIDs to achieve symptom resolution by day 3 or 4 post-randomization in one additional person as the reciprocal of the difference in risk, or 1/(absolute risk reduction). Because of clinical heterogeneity in antibiotic exposure, NSAID selection, and outcomes reported in included studies, we did not conduct formal meta-analyses.

## RESULTS

### Search Results and Study Details

**Fig. 1:**
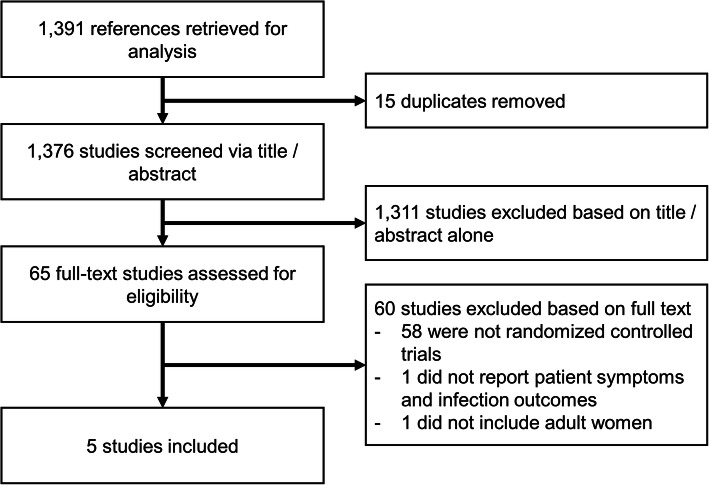
Flow diagram of study selection.

A total of 1391 citations were retrieved by our search, including 15 duplicates removed prior to screening. Of these, 65 full-text studies were eligible for inclusion following title and abstract review and 5 randomized clinical trials were included (Fig. 1). Interrater agreement for study inclusion was excellent at 0.97. Four studies were conducted in Europe, and one was conducted in Pakistan.

### Characteristics of Included Patients

Four studies^22, 23, 28, 29^ included adult women over the age of 18 while one study^30^ included women over the age of 15. Upper age limits ranged from 50 to 70 years, with one study^22^ having no upper age limit (Table 1). UTIs were generally defined as a constellation of typical symptoms (i.e., dysuria, frequency, urgency, and suprapubic/lower abdominal pain). One study^28^ included individuals with self-diagnosed symptomatic cystitis if they had urine dipstick results positive for nitrites, leukocyte esterase, or both. Four studies^22, 23, 28, 29^ excluded patients with upper UTI signs (i.e., fever and back pain), pregnancy, diabetes, prior urological interventions, immunosuppressive states, urinary catheterization, recent UTI, current antibiotic or NSAID use, or contraindications to study drugs. Four studies^22, 23, 28, 29^ included baseline urine culture results at the time of inclusion, though culture results were not used as eligibility criteria (Table 1). The share of patients with positive urine cultures at the time of inclusion ranged from 67 to 86% in the NSAID groups and 64 to 80% in the antibiotic groups (Table 1).

**Table 1:** Characteristics of Included Randomized Controlled Trials

Study, year	Country	Publication status	Patients, *n*		Definition of UTI	Patients with positive urine cultures, *n* (%)*		Gender and age criteria	Follow-up period, days	Medication regimen		Risk of bias^†^
			NSAID	Antibiotic		NSAID	Antibiotic			NSAID	Antibiotic	
Bleidorn et al., 2010	Germany	Full text	40	39	Typical symptoms of a UTI (dysuria and/or frequency)	31 (86)	24 (80)	Women > 18 years	28	Ibuprofen 400 mg 3 × per day for 3 days	Ciprofloxacin 250 mg 2 × per day (+ 1 placebo) for 3 days	Unclear
Gágyor et al., 2015	Germany	Full text	248	246	Dysuria and/or frequency/urgency of micturition, with or without lower abdominal pain	179 (76)	181 (77)	Women 18–65 years	28	Ibuprofen 400 mg 3x per day for 3 days plus 1 sachet placebo granules	Fosfomycin-trometamol 3 g sachet × 1 plus placebo tablets 3 × per day for 3 days	Low
Jamil et al., 2016	Pakistan	Full text	50	50	Symptoms of urinary frequency, urgency, dysuria, and suprapubic pain associated with UTI	NR	NR	Women 15–50 years	5	Potassium citrate 2 × per day + Flurbiprofen 100 mg 2 × per day for 5 days	Ciprofloxacin 250 mg 2 × per day for 5 days	High
Kronenberg et al., 2017	Switzerland	Full text	133	120	One or more symptoms of typical lower UTI (dysuria, frequency, macrohematuria, cloudy or smelly urine) or self-diagnosed symptomatic cystitis if urine dipstick positive for nitrites, leukocytes, or both	96 (72)	89 (74)	Women 18–70 years	30	Diclofenac 75 mg 2 × per day for 3 days	Norfloxacin 400 mg 2 × per day for 3 days	Low
Vik et al., 2018	Norway, Denmark, and Sweden	Full text	194	189	Dysuria combined with either increased urinary frequency or urgency or both, with or without visible hematuria	121 (67)	113 (64)	Women 18–60 years	28	Ibuprofen 600 mg 3 × per day for 3 days	Pivmecillinam 200 mg 3 × per day for 5 days	Low

*NSAID*, non-steroidal anti-inflammatory drug; *UTI*, urinary tract infection; *NR*; not reported

*Percentage of patients with a “positive culture” at the time of enrollment of those with reported urine culture results. The cutoff for identifying “positive cultures” varied between studies from ≥ 10^2^ colony forming units/mL to ≥ 10^5^ colony forming units/mL

†Assessed using the Cochrane Collaboration tool

### Study Medications

All studies compared the use of NSAIDs with antibiotic therapy for the treatment of uncomplicated UTIs (Table 1). One study^30^ used potassium citrate in addition to NSAID therapy in the treatment arm. The NSAID was ibuprofen in three trials,^22, 23, 29^ flurbiprofen in one trial,^30^ and diclofenac in one trial.^28^ Antibiotic therapy also varied across studies with two studies^22, 30^ using ciprofloxacin, one study^23^ using fosfomycin-trometamol, one study^28^ using norfloxacin, and one study^29^ using pivmecillinam.

### Symptom Resolution Outcomes

Though all studies included symptom resolution as a primary outcome, the method of assessment differed (Table 2). Symptom resolution was assessed as follows: percent of patients with symptom resolution on day 3 (one study^28^); percent of patients with symptom resolution on day 4 (two studies^22, 29^); symptom burden on days 0–7 measured as area under the curve of the sum of daily symptom scores (one study^23^); and comparison of pre- and post-treatment symptom scores on day 5 (one study^30^). For these endpoints, two studies tested for non-inferiority only, using non-inferiority margins of 10%^23^ and 25%,^29^ one tested for non-inferiority (using a 15% non-inferiority margin) and superiority,^28^ and two tested for differences between the two groups^22, 30^. The results for these primary outcomes are presented in Table 2.

**Table 2:** Primary and Secondary Outcomes of Included Randomized Controlled Trials

Study, year	Primary outcomes		Secondary outcomes
	Measure	Findings	
Bleidorn et al., 2010	Symptom resolution by day 4 (test for difference)	No significant difference between NSAIDs and antibiotics in achieving symptom resolution by day 4	Burden of symptoms, frequency of relapses, secondary antibiotic treatments, incidence of adverse events
Gágyor et al., 2015	Burden of symptoms on days 0–7, measured as AUC of the sums of daily symptom scores (test for non-inferiority); number of courses of antibiotics on days 0–28 (test for superiority)	NSAIDs inferior to antibiotics in achieving symptom resolution as measured by the ratio of the AUC of the sum of symptom scores on days 0–7 for both groups	Number of adverse events (including pyelonephritis), frequency of relapses, symptom burden, number of antibiotic doses per patient
Jamil et al., 2016	Comparison of post-treatment total symptom scores on day 5 (test for difference)	No significant difference between symptom scores on day 5 for NSAID and antibiotic groups	Comparison of scores for specific symptoms (frequency, urgency, dysuria, suprapubic pain)
Kronenberg et al., 2017	Symptom resolution by day 3 (test for non-inferiority and superiority)	NSAIDs inferior to antibiotics in achieving symptom resolution and antibiotics superior to NSAIDs in achieving symptom resolution by day 3	Use of antibiotics up to day 30, re-consultation for UTIs, mean composite symptom scores, adverse events (including pyelonephritis), time to resolution of symptoms, working days lost, satisfaction with management
Vik et al., 2018	Symptom resolution by day 4 (test for non-inferiority)	NSAIDs inferior to antibiotics in achieving symptom resolution by day 4	Duration of symptoms and symptom load; proportion of patients with a positive second urine culture, in need of a medical consult for UTI within 4 weeks, and who received antibiotics during the study period; frequency of adverse events (including pyelonephritis)

*NSAID*, non-steroidal anti-inflammatory drug; *UTI*, urinary tract infection; *AUC*, area under the curve

**Fig. 2:**
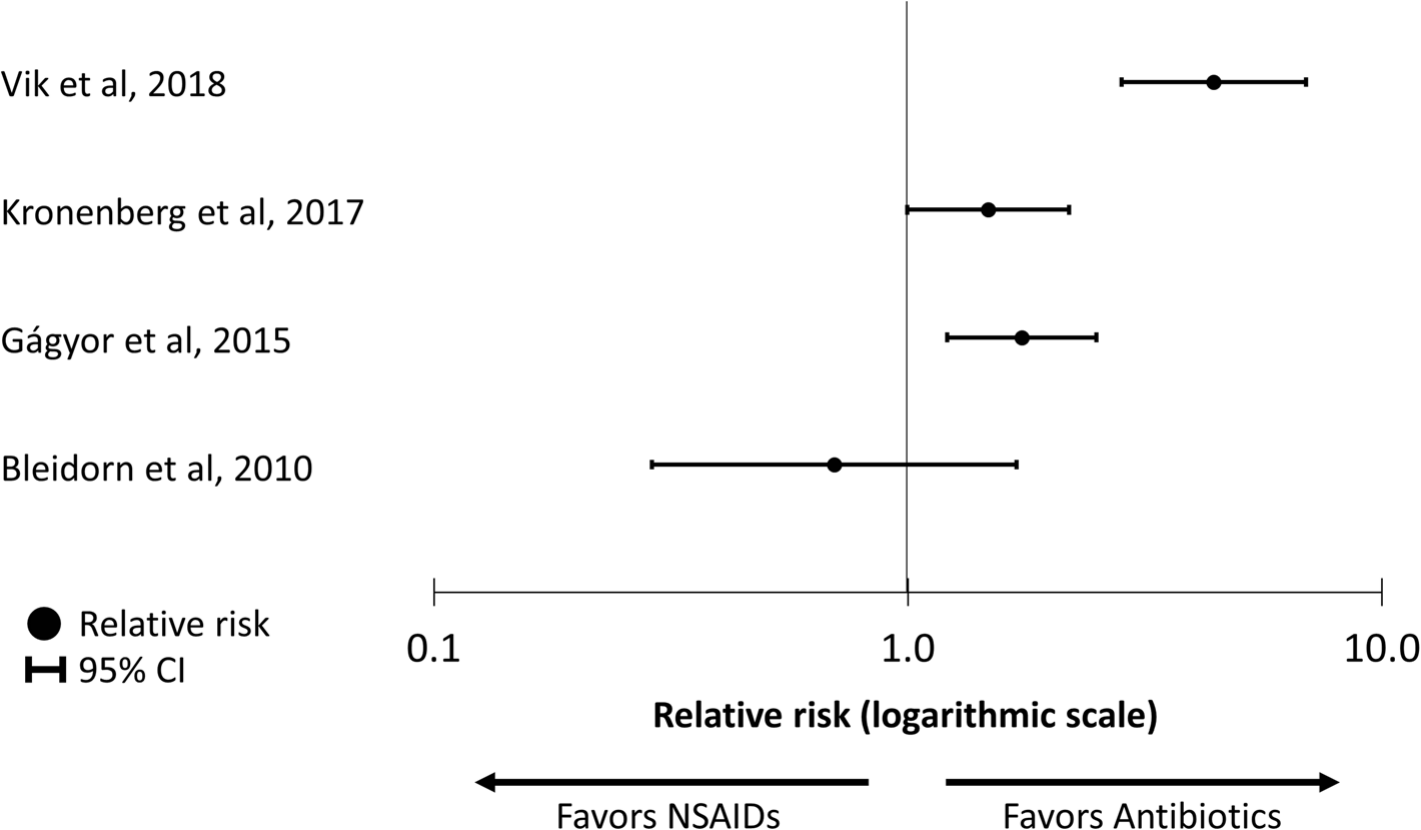
Comparison of relative risk of symptom resolution by day 4 post-randomization for patients treated with non-steroidal anti-inflammatory drugs (NSAIDs) versus antibiotics.

Of the studies reporting symptom resolution by day 3 or 4 post-randomization (all low risk of bias), one smaller study^22^ (79 patients) demonstrated no significant difference between NSAIDs and antibiotics for symptom resolution (58% of patients treated with NSAIDs had symptoms resolution at 3 days versus 52% of those treated with antibiotics, *p* = 0.744 for difference). Three larger studies^23, 28, 29^ (1130 total patients) demonstrated higher rates of symptom resolution in the antibiotic groups, with symptom resolution 17 to 35 percentage points higher in the antibiotic group compared with the NSAID group (Table 3; Fig. 2). One study^30^ (high risk of bias) compared symptom scores at the end of the trial, day 5 post-randomization, and found no significant difference in scores between the NSAID and antibiotic groups (1.4 versus 1.9; *p* = 0.13 for difference). Table 4 shows the NNT with antibiotics to achieve symptom resolution in one additional patient by days 3 to 4 post-randomization, which range from 3.0^29^ to 6.4.^23^

**Table 3:** Summary of Reported Outcomes in Randomized Controlled Trials Assessing NSAIDs Versus Antibiotics for Women with Uncomplicated UTIs

	Patients, *n*		Patients with symptom resolution by day 3 or 4, *n* (%)			Mean of post-treatment total symptom score on day 5*			Women receiving antibiotics for any reason during study period, *n* (%)			Patients with pyelonephritis during the study period, *n* (%)		
Study, year	NSAID	Antibiotic	NSAID	Antibiotic	Risk difference (95% CI)^†^	NSAID	Antibiotic	*p* value	NSAID	Antibiotic	Risk difference (95% CI)^‡^	NSAID	Antibiotic	Risk difference (95% CI)^§^
Bleidorn et al., 2010	40	39	21 (58)^|^	17 (52)^|^	9 (− 13 to 31)^|^	NR	NR	NR	NR	NR	NR	NR	NR	NR
Gágyor et al., 2015	248	246	91 (39)^|^	129 (56)^|^	17 (9 to 26)^|^	NR	NR	NR	85 (35)	243 (100)	− 65 (− 71 to − 59)	5 (2)	1 (0.4)	1.7 (− 0.3 to 3.6)
Jamil et al., 2016	50	50	NR	NR	NR	1.4	1.9	0.13	NR	NR	NR	NR	NR	NR
Kronenberg et al., 2017	133	120	72 (54)^¶^	96 (80)^¶^	27 (15 to 38)^¶^	NR	NR	NR	82 (62)	118 (98)	− 37 (− 46 to − 28)	6 (5)	0 (0)	5 (1 to 8)
Vik et al., 2018	194	189	70 (39)^|^	131 (74)^|^	35 (27 to 43)^|^	NR	NR	NR	NR	NR	NR	7 (4)	0 (0)	4 (1 to 8)

*UTI*, urinary tract infection; *NSAID*, non-steroidal anti-inflammatory drug; *NR*, not reported

*Total symptom score is the sum of symptom scores for urinary frequency, urgency, dysuria, and pain

†Positive numbers indicate higher rates of symptom resolution among patients receiving antibiotics compared with those receiving NSAIDS

‡Positive numbers indicate higher rates of antibiotic use in the NSAID group

^§^Positive numbers indicate higher rates of pyelonephritis in the NSAID group

^|^Symptom resolution by day 4

^¶^Symptom resolution by day 3

**Table 4:** Number Needed to Treat with Antibiotics Rather than Non-steroidal Anti-inflammatory Drugs (NSAIDs) to Achieve Symptom Resolution in One Additional Person by Day 3 or 4 or Prevent One Additional Case of Pyelonephritis by Day 28 to 30

Study*	NNT to achieve symptom resolution in one additional patient by day 3 or 4^†^	NNT to prevent one additional case of pyelonephritis by days 28 to 30^‡^
Gágyor et al., 2015	6.4	62.1
Kronenberg et al., 2017	3.9	22.2
Vik et al., 2018	3.0	27.7

*NNT*, number needed to treat

*Bleidorn et al. (2010)^22^ was not included as it demonstrated higher symptom resolution in the NSAID group compared with the antibiotic group and did not report subsequent cases of pyelonephritis. Jamil et al. (2016)^30^ was not included as it did not report the number of individuals with symptom resolution by day 3 or 4 and did not report subsequent cases of pyelonephritis

†Number needed to treat to achieve additional symptom resolution calculated for three trials that included symptom resolution by day 3 or 4 as a primary or secondary outcome and there was a statistically significant difference in symptom resolution between the NSAID and antibiotic groups

‡Number needed to treat to prevent one additional case of pyelonephritis calculated for three trials that reported incidence of pyelonephritis in the study period

### Secondary Outcomes

Two studies^23, 28^ reported the proportion of participants that received antibiotics for any reason during the study period—both studies reported fewer antibiotic prescriptions in the NSAID versus antibiotic groups (Table 3). Three studies^23, 28, 29^ reported rates of pyelonephritis during the trial period. Among these, pyelonephritis was uncommon, occurring in fewer than 5% of patients in any arm. Two studies found patients who received antibiotics had lower rates of pyelonephritis compared with those who received NSAIDs. Table 4 shows the number of individuals who would need to be treated with antibiotics over NSAIDs to avoid one case of pyelonephritis.

### Risk of Bias

Three of the studies were found to have a low risk of bias, one was found to have an unclear risk of bias, and one was found to have a high risk of bias (Table 1).

## DISCUSSION

In this systematic review, we evaluated five randomized controlled trials (of 1309 patients) comparing NSAIDs with antibiotics for treatment of uncomplicated UTI. Although study protocols and reported outcomes were heterogeneous, two smaller studies that were at high and unclear risk of bias found no difference between NSAIDs and antibiotics, while three studies with low risk of bias supported antibiotic therapy over NSAIDs in terms of symptom resolution. Furthermore, two studies at low risk of bias found higher risks of pyelonephritis in patients who received NSAIDs versus antibiotics; the third study with low risk of bias also found a higher risk of pyelonephritis in patients receiving NSAIDs, but the confidence interval for the risk difference included zero. In sum, these findings support the use of antibiotics as first-line treatment for uncomplicated UTI for both symptom resolution and prevention of pyelonephritis.

Despite the superiority of antibiotic therapy, 39–58% of participants in the NSAID groups achieved symptom resolution by day 3 or 4.^22, 23, 28, 29^ For comparison, a meta-analysis of placebo-controlled trials demonstrated clinical success (symptom improvement or resolution by first follow-up visit) in 25–54% of participants in the placebo arms.^13, 25^ One possible explanation for why many UTI symptoms resolved without antibiotic therapy is that not all women included in the studies may have had true UTIs. Inclusion in most studies was based on symptoms alone—a commonly accepted approach for uncomplicated UTIs^31^ that is consistent with several clinical practice guidelines from countries where the trials were conducted.^32–34^ However, clinical symptoms only correctly diagnose UTIs half of the time.^31, 35^ Thus, it is possible that a large number of participants may not have had a UTI or been at risk of worsening infection. Indeed, in the four studies that collected urine specimens for culture at the time of inclusion, only 64–86% of patients in either study arm had positive cultures.^22, 23, 28, 29^ Women often suffer from urinary symptoms for a host of reasons (e.g., incontinence, medication side effects) that are misdiagnosed as a UTI.^36^ Barriers to obtaining timely urine testing often promote alternatives to testing, such as “as needed” antibiotic prescriptions or prescriptions by phone.^37^

Furthermore, even patients with “positive” urine cultures may have asymptomatic bacteriuria and thus not require treatment. Misdiagnosis of UTI is particularly common in elderly women who have other long-standing urinary tract symptoms, such as incontinence.^38–40^ However, the maximum age cutoffs used in four out of five studies (50 to 70 years of age) likely avoided some challenges of diagnosing UTIs in the elderly—while also limiting generalizability to these groups. In summary, although there is a subset of individuals who clearly benefit from antibiotics, there is likely another segment that does not benefit from antibiotics and could be appropriately treated with NSAIDs or no therapy.

Another important finding from the included studies was that only 2.0–4.5% of individuals treated with NSAIDs subsequently developed pyelonephritis (based on 3 studies with 1130 patients).^23, 28, 29^ Thus, to avoid one case of pyelonephritis, 22 to 62 people would need to be treated with antibiotics. Notably, these reported rates of pyelonephritis in NSAID groups were higher than placebo arms of prior clinical trials (0.4%^41^ and 2.6%^12^). While better follow-up to assess outcomes in the NSAID studies may be partially responsible, NSAIDs have also been associated with worse outcomes in other infections, such as community-acquired pneumonia,^42, 43^ possibly due to immune suppresion.^44^ These and other potential adverse effects of NSAIDs must be better understood before these drugs can be used for uncomplicated UTI.

In summary, this review demonstrates the superiority of antibiotics over NSAIDs for the treatment of uncomplicated UTIs. However, future research should focus on better diagnosing UTI and stratifying women with uncomplicated UTIs to identify the subset that would benefit from antibiotic treatment while avoiding unnecessary antibiotic prescriptions. Other potential antibiotic-sparing strategies should be further explored as well, including delayed antibiotic prescriptions for lingering symptoms only,^37^ point-of-care testing–driven prescribing algorithms,^45^ and provider audit reports and reminders.^46^ Given the high incidence of uncomplicated UTIs (and likely misdiagnosed UTIs) as well as the number of antibiotic prescriptions written for these infections,^1, 2, 4, 47^ elimination of even a quarter of antibiotic prescriptions for uncomplicated UTIs would result in tens of thousands fewer antibiotic courses per year. Such a shift in practice patterns could have significant downstream ramifications, including reducing the number of adverse drug reactions, *Clostridium difficile* infections, and multi-drug resistant organisms.^48^

This review has several strengths, including a comprehensive literature search that included multiple databases without language filters as well as a published protocol. It also has several limitations. We could not perform a meta-analysis given substantial heterogeneity in treatment across studies. Second, there was substantial variability in reported outcomes. Third, only five trials met inclusion criteria, limiting the ability to detect rare outcomes. Fourth, the follow-up period for all studies was 1 month or less, preventing the assessment of long-term impact of NSAID versus antibiotic therapy on patient outcomes, though a follow-up study to one trial demonstrated no impact on recurrent UTI or pyelonephritis from 28 days to 6 months post-randomization.^49^

## CONCLUSION

This review adds two important pieces of information to what is currently known about UTI. First, it confirms the role of antibiotics as first-line treatment for uncomplicated UTIs compared with symptomatic treatment with NSAIDs. Second, it demonstrates that about half of patients experience symptom resolution with NSAIDs alone, indicating a potential target to reduce antibiotic prescriptions. While fewer than 5% of those using NSAIDs developed pyelonephritis, the potential adverse effects of NSAIDs as treatment need to be considered. Taken together, these data point to the importance of future research to identify patients who would most benefit from antibiotic therapy so that prescribing can be targeted towards those individuals while avoiding prescribing to those who would not derive incremental benefit. Such an approach would ensure women have adequate, prompt symptom resolution while at the same time avoiding unnecessary antibiotic prescribing—the kind of “win-win” that is the ultimate goal of antibiotic stewardship efforts.

## Electronic supplementary material

ESM 1 (DOC 63 kb)

ESM 2 (DOCX 29 kb)
