# Supplementary material for: Is Non-Steroidal Anti-Inflammatory Therapy Non-Inferior to Antibiotic Therapy in Uncomplicated Urinary Tract Infections: a Systematic Review
Source: J Gen Intern Med. 2020 Apr 8;35(6):1821–9. doi: 10.1007/s11606-020-05745-x (PMC7280390; doi:10.1007/s11606-020-05745-x)
Supplement: Supplementary file 2 — (DOCX 29 kb) [file 11606_2020_5745_MOESM2_ESM.docx]

# Appendix: Detailed search strategy

All searches run from inception of database through January 13, 2020.

No limits applied.

## Search strategy by database

1. **PubMed -** (((“Urinary Tract Infections”[mesh] OR “Urinary tract infection”[tw] OR “Urinary tract infections”[tw] OR UTI[tw] OR UTIs[tw])) AND (“Analgesics”[mesh] OR analgesic[tw] OR analgesics[tw] OR Anodynes[tw] OR anodyne[tw] OR Ibuprofen[tw] OR Nsaid[tw] OR nsaids[tw])) AND (“Anti-bacterial Agents”[mesh] OR “Anti-infective agents, urinary”[mesh] OR antimicrobial[tw] OR antimicrobials[tw] OR Antibiotic[tw] OR Antibiotics[tw] OR Nitrofurantoin[tw] OR Trimethoprim*[tw] OR TMP-SMX[tw] OR Co-trimoxazole[tw] OR fosfomycin[tw] OR Pivmecillinam[tw] OR Fluoroquinolone*[tw] OR Ofloxacin[tw] OR Ciprofloxacin[tw] OR Levofloxacin[tw] OR Norfloxacin[tw] OR Amoxicillin[tw] OR Cefdinir[tw] OR Cefaclor[tw] OR Cefpodoxime[tw] OR Cephalexin[tw] OR Ampicillin[tw] OR Beta-lactam*[tw] OR “nalidixic acid”[tw] OR quinolone*[tw] OR Sparfloxacin[tw] OR Lomefloxacin[tw] OR Ofloxacin[tw] OR Rufloxacin[tw] OR Pefloxacin[tw])
2. **Embase.com -** ((('Urinary Tract Infection'/exp/mj OR "Urinary tract infection":ti,ab OR "Urinary tract infections":ti,ab OR UTI:ti,ab OR UTIs:ti,ab)) AND ('Analgesic Agent'/exp OR analgesic:ti,ab,de,tn OR analgesics:ti,ab,de,tn OR Anodynes:ti,ab,de,tn OR anodyne:ti,ab,de,tn OR Ibuprofen:ti,ab,de,tn OR Nsaid:ti,ab,de,tn OR nsaids:ti,ab,de,tn)) AND ('Antibiotic  Agent'/exp OR 'Urinary Tract Antiinfective Agent'/exp OR antimicrobial:ti,ab,de,tn OR antimicrobials:ti,ab,de,tn OR Antibiotic:ti,ab,de,tn OR Antibiotics:ti,ab,de,tn OR Nitrofurantoin:ti,ab,de,tn OR Trimethoprim*:ti,ab,de,tn OR TMP-SMX:ti,ab,de,tn OR Co-trimoxazole:ti,ab,de,tn OR fosfomycin:ti,ab,de,tn OR Pivmecillinam:ti,ab,de,tn OR Fluoroquinolone*:ti,ab,de,tn OR Ofloxacin:ti,ab,de,tn OR Ciprofloxacin:ti,ab,de,tn OR Levofloxacin:ti,ab,de,tn OR Norfloxacin:ti,ab,de,tn OR Amoxicillin:ti,ab,de,tn OR Cefdinir:ti,ab,de,tn OR Cefaclor:ti,ab,de,tn OR Cefpodoxime:ti,ab,de,tn OR Cephalexin:ti,ab,de,tn OR Ampicillin:ti,ab,de,tn OR Beta-lactam*:ti,ab,de,tn OR "nalidixic acid":ti,ab,de,tn OR quinolone*:ti,ab,de,tn OR Sparfloxacin:ti,ab,de,tn OR Lomefloxacin:ti,ab,de,tn OR Ofloxacin:ti,ab,de,tn OR Rufloxacin:ti,ab,de,tn OR Pefloxacin:ti,ab,de,tn)
3. **Scopus -** (((INDEXTERMS("Urinary Tract Infections") OR TITLE-ABS-KEY("Urinary tract infection") OR TITLE-ABS-KEY("Urinary tract infections") OR TITLE-ABS-KEY("UTI") OR TITLE-ABS-KEY("UTIs"))) AND (INDEXTERMS("Analgesics") OR TITLE-ABS-KEY("analgesic") OR TITLE-ABS-KEY("analgesics") OR TITLE-ABS-KEY("Anodynes") OR TITLE-ABS-KEY("anodyne") OR TITLE-ABS-KEY("Ibuprofen") OR TITLE-ABS-KEY("Nsaid") OR TITLE-ABS-KEY("nsaids") OR TITLE-ABS-KEY("non-steroidal"))) AND (INDEXTERMS("Anti-bacterial Agents") OR INDEXTERMS("Anti-infective agents, urinary") OR TITLE-ABS-KEY("antimicrobial") OR TITLE-ABS-KEY("antimicrobials") OR TITLE-ABS-KEY("Antibiotic") OR TITLE-ABS-KEY("Antibiotics") OR TITLE-ABS-KEY("Nitrofurantoin") OR TITLE-ABS-KEY("Trimethoprim*") OR TITLE-ABS-KEY("TMP-SMX") OR TITLE-ABS-KEY("Co-trimoxazole") OR TITLE-ABS-KEY("fosfomycin") OR TITLE-ABS-KEY("Pivmecillinam") OR TITLE-ABS-KEY("Fluoroquinolone*") OR TITLE-ABS-KEY("Ofloxacin") OR TITLE-ABS-KEY("Ciprofloxacin") OR TITLE-ABS-KEY("Levofloxacin") OR TITLE-ABS-KEY("Norfloxacin") OR TITLE-ABS-KEY("Amoxicillin") OR TITLE-ABS-KEY("Cefdinir") OR TITLE-ABS-KEY("Cefaclor") OR TITLE-ABS-KEY("Cefpodoxime") OR TITLE-ABS-KEY("Cephalexin") OR TITLE-ABS-KEY("Ampicillin") OR TITLE-ABS-KEY("Beta-lactam*") OR TITLE-ABS-KEY("nalidixic acid") OR TITLE-ABS-KEY("quinolone*") OR TITLE-ABS-KEY("Sparfloxacin") OR TITLE-ABS-KEY("Lomefloxacin") OR TITLE-ABS-KEY("Ofloxacin") OR TITLE-ABS-KEY("Rufloxacin") OR TITLE-ABS-KEY("Pefloxacin"))
4. **Web of Science Core Collection -** TOPIC: ("Urinary Tract Infections" OR "Urinary tract infection" OR "Urinary tract infections" OR UTI OR UTIs) *AND* TOPIC: (Analgesics OR analgesic OR analgesics OR Anodynes OR anodyne OR Ibuprofen OR Nsaid OR nsaids OR non-steroidal OR nonsteroidal) *AND* TOPIC: ("Anti-bacterial Agents" OR "Anti-infective agents, urinary" OR antimicrobial OR antimicrobials OR Antibiotic OR Antibiotics OR Nitrofurantoin OR Trimethoprim* OR TMP-SMX OR Co-trimoxazole OR fosfomycin OR Pivmecillinam OR Fluoroquinolone* OR Ofloxacin OR Ciprofloxacin OR Levofloxacin OR Norfloxacin OR Amoxicillin OR Cefdinir OR Cefaclor OR Cefpodoxime OR Cephalexin OR Ampicillin OR Beta-lactam* OR "nalidixic acid" OR quinolone* OR Sparfloxacin OR Lomefloxacin OR Ofloxacin OR Rufloxacin OR Pefloxacin)
5. **CINAHL -** ((MH "Urinary Tract Infections+") OR "Urinary tract infection" OR "Urinary tract infections" OR UTI OR UTIs) AND ((MH "Analgesics+") OR analgesic OR analgesics OR Anodynes OR anodyne OR Ibuprofen OR Nsaid OR nsaids) AND ((MH "Anti-bacterial Agents+") OR (MH "Anti-infective agents, urinary+") OR antimicrobial OR antimicrobials OR Antibiotic OR Antibiotics OR Nitrofurantoin OR Trimethoprim* OR TMP-SMX OR Co-trimoxazole OR fosfomycin OR Pivmecillinam OR Fluoroquinolone* OR Ofloxacin OR Ciprofloxacin OR Levofloxacin OR Norfloxacin OR Amoxicillin OR Cefdinir OR Cefaclor OR Cefpodoxime OR Cephalexin OR Ampicillin OR Beta-lactam* OR "nalidixic acid" OR quinolone* OR Sparfloxacin OR Lomefloxacin OR Ofloxacin OR Rufloxacin OR Pefloxacin)
6. **ClinicalTrials.gov** - Condition or Disease: Urinary Tract Infections; Other terms:(antibiotics OR antibiotic OR antimicrobial OR Nitrofurantoin OR Trimethoprim OR TMP-SMX OR Co-trimoxazole OR fosfomycin OR Pivmecillinam OR Fluoroquinolone OR Ofloxacin OR Ciproflox) AND (analgesics OR analgesic OR NSAIDs OR NSAID OR nonsteroidal)

# 
